# Chronic brain inflammation causes a reduction in GluN2A and GluN2B subunits of NMDA receptors and an increase in the phosphorylation of mitogen-activated protein kinases in the hippocampus

**DOI:** 10.1186/1756-6606-7-33

**Published:** 2014-04-24

**Authors:** Jinhua Ma, Bo-Ryoung Choi, ChiHye Chung, Sun Seek Min, Won Kyung Jeon, Jung-Soo Han

**Affiliations:** 1 Department of Biological Sciences, Konkuk University, 120 Neungdong-ro, Gwangjin-gu, Seoul 143-701, Republic of Korea; 2 Department of Physiology, Eulji University, College of Medicine, Daejeon 301-832, Republic of Korea; 3 Traditional Korean Medicine Information Research Division, Korea Institute of Oriental Medicine, Daejeon 305-811, Republic of Korea

**Keywords:** Neuroinflammation, Memory, NMDA, MAPK, Microglia

## Abstract

Neuroinflammation plays a key role in the initiation and progression of neurodegeneration in Alzheimer’s disease (AD). Chronic neuroinflammation results in diminished synaptic plasticity and loss of GluN1 *N*-methyl-D-aspartate (NMDA) receptors in the hippocampus, leading to the cognitive deficits that are the most common symptoms of AD. Therefore, it is suggested that chronic inflammation may alter expression levels of GluN2A and GluN2B subunits of NMDA receptors and associated intracellular signalling. Chronic neuroinflammation was induced by chronic infusion of lipopolysaccharide (LPS) into the fourth ventricle in Fischer-344 rats. The status of hippocampus-dependent memory was evaluated in control rats and rats chronically infused with LPS. Microglial activation in the hippocampus was examined using immunohistochemical staining. Western blot analysis was used to measure membrane levels of GluN2A and GluN2B subunits of NMDA receptors and mitogen-activated protein kinase (MAPK) in the hippocampi of these rats, and immunofluorescent double labeling was used to assess the cellular location of MAPK. Microglial activation was observed in the hippocampi of rats that showed memory impairments with chronic LPS infusion. Chronic LPS infusion reduced the levels of GluN2A and GluN2B and increased the levels of phosphorylated MAPKs in the hippocampus. MAPK-positive immunoreactivity was observed mostly in the neurons and also in non-neuronal cells. Reductions in GluN2A and GluN2B subunits of NMDA receptors coupled with altered MAPK signaling, in response to inflammatory stimuli may be related to the cognitive deficits observed in AD.

## Background

Neuroinflammation plays a key role in the initiation and progression of neurodegeneration in Alzheimer’s disease (AD) [1-5]. *In vivo* measurement of activated microglia using brain imaging suggests that inflammation is an early event in the pathogenesis of the disease [6,7]. A series of animal experiments with chronic lipopolysaccharide (LPS) infusion into the brain has demonstrated that inflammation leads to cognitive deficits, which are the most common symptom of AD [8,9].

It has also been demonstrated that brain inflammation leads to a decrease in the number of GluN1 *N*-methyl-*D*-aspartate receptors (NMDARs) within the dentate gyrus (DG) and cornu ammonis 3 (CA3) hippocampal areas, without any loss of neurons [10]. On the basis of the association of GluN1 and synaptic plasticity [11], electrophysiological studies were performed to examine the effects of chronic LPS infusion on long-term potentiation (LTP) and long-term depression (LTD) in the hippocampus [12-14]. LPS infusion has been shown to impair LTP in perforant path-granular cell synapses [12]. In addition, voltage-dependent calcium channel dependent-LTP and NMDA receptor (NMDAR)-dependent LTP has been attenuated [13] as well as LTD has been impaired [14] in Schaffer collateral-cornu ammonis 1 (CA1) synapses in the hippocampus of rats chronically infused with LPS.

NMDARs are tetrameric protein complexes composed of GluN1 subunits with at least one type of GluN2 subunit. As stated above, chronic LPS infusion impairs synaptic plasticity and reduces the number of GluN1 NMDARs. Therefore, it is predicted that chronic LPS infusion would reduce the expression levels of GluN2A and GluN2B subunits of NMDARs, which are essential mediators of synaptic plasticity [15]. Studies using primary cultured neurons have reported that in mature neurons, GluN2B is associated with inhibition, rather than activation, of extracellular signal-regulated kinases 1/2 (Erk1/2) that are the best studied mitogen-activated protein kinases (MAPKs) [16]. It has also been shown that the phosphorylated levels of p38, a subfamily of MAPKs, depend on the concentration of experimentally applied NMDA and NMDAR subtype composition or localization [17].

In addition, in neurological disorders such as AD, it is well known that MAPKs are crucial for the regulation of microglia-mediated inflammation [18] and are key regulators of tau phosphorylation and plaque formation, which can eventually lead to dementia and AD [19]. Our previous study reported increased levels of phosphorylated Erk1/2 (p-Erk1/2) in the hippocampus following microglial activation by chronic LPS infusion [20]. The present experiment was conducted to evaluate the effects of chronic LPS infusion on the expression levels of GluN2A and GluN2B subunits of NMDARs and its associated Erk and p38 signaling. In the present study, membrane levels of GluN2A and GluN2B were reduced in the hippocampi of Fischer-344 rats that exhibited neuroinflammatory responses and memory impairments induced by chronic LPS infusion into the 4^th^ ventricle. In addition, levels of p-Erk1/2 and phosphorylated p38 (p-p38) were increased in the hippocampus of chronically LPS-infused rats, and these immunoreactivities were observed mostly in neurons and also in non-neuronal cells in the hippocampi of LPS-infused rats.

## Results and discussion

### Spatial memory impairments in chronically LPS-infused rats

**Figure 1:**
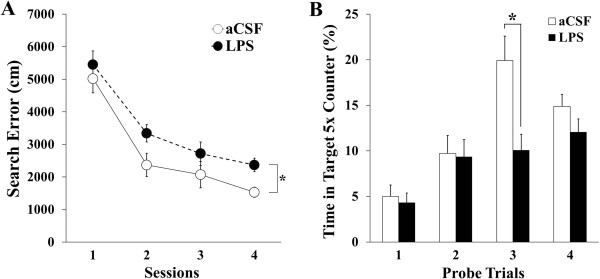
**Performance of aCSF- and LPS-infused rats in the spatial version of a Morris water maze. (A)** Search error in finding a hidden platform in a spatial learning task during 5 training trial blocks. The artificial cerebrospinal fluid (aCSF)-infused rats (labeled as aCSF) became proficient at locating the submerged platform during training. The lipopolysaccharide (LPS)-infused rats (labeled as LPS) showed less improvement over the course of training when compared with the aCSF-infused rats (*, *p* < 0.05). **(B)** The percentage of time spent in the target annulus which was 5 times of the platform size, during the 30 s probe trial. The aCSF-infused rats spent more time in the target area than the LPS-infused rats, and this was statistically significant at the third probe trial (*, *p* < 0.05).

As reported previously, LPS-infused rats showed impairments of spatial memory [12,13]. The average search error in the spatial learning task during a 5- training trial block was used to assess spatial memory in the rats. The artificial cerebrospinal fluid (aCSF) -infused rats became proficient at locating the submerged platform during the training trials. Compared with the aCSF-infused rats, the LPS-infused rats showed less improvement over the course of the training, evidenced by that acquisition slope in the spatial learning differed in two groups (F (1, 21) = 4.67, *p* < 0.05, Figure 1A). The one-way analysis of variance (ANOVA) with repeated variable for search error showed that the between-group effects (aCSF vs. LPS) were significant (F (1, 21) = 5.47, *p* < 0.05) and the training effects were also significant (F (3, 63) = 47.81, *p* < 0.001). There were no interaction effects of group and training (F (3, 63) = 0.31, no significance [ns]).

The percentage of time spent in a target area containing the platform and 5 times of the platform diameter was measured during the 30 s probe trial (Figure 1B). The repeated ANOVA showed that the between-group effects were significant (F (1, 21) = 4.80, *p* < 0.05), the spatial bias over the course of training was significantly increased (F (3, 63) = 18.41, *p* < 0.01), and the interaction effects of the group and probe were significant (F (3, 63) = 4.20, *p* < 0.01). A post-hoc analysis revealed spatial bias in the probe trial conducted 24 h after 3 blocks of training trials (15 trials) was higher in the aCSF-infused rats than in the LPS-infused rats (*p* < 0.05). There were no differences in swimming speeds between the 2 groups of rats (data not shown).

### Activated microglia in the hippocampus of chronically LPS-infused rats

The locations of the cannula tips were verified while sectioning the brain portions containing the 4^th^ ventricle using the microtome. All cannula tips were located in the 4^th^ ventricle. Tissues from 17 rats were used for immunohistological observations and those from the remaining 6 rats for western blot analyses.

Among brain cells, ionized calcium binding adaptor molecule 1 (Iba-1) expression is restricted in ramified microglia/macrophage [21] and its expression is up-regulated in activated microglial/macrophage [22] following exposure to an inflammatory stimulus or an inflammation-induced challenge such as facial nerve axotomy [23]. Compared to the aCSF-infused rats, the LPS-infused rats showed more microglial cells in the hippocampal CA1, CA3, and DG areas (Additional file 1: Figure S1). The repeated ANOVA showed that the between-group effects (aCSF vs. LPS) were significant (F (1, 6) = 6.50, *p* < 0.05), whereas the regional differences (CA1, CA3, and DG) effects were not significant (F (2, 12) = 3.34, ns). There were no interaction effects of group and region (F (2, 12) = 1.26, ns).

In the present study, we also observed activated microglia with immunostaining for OX-6 directed against the Class II major histocompatibility complex. Activated microglial cells were highly distributed in hippocampal CA3 and DG areas in the LPS-infused rats (Additional file 1: Figure S1). In contrast, aCSF-infused rats had no activated microglial cells. The OX-6-positive microglial cells within the hippocampus were counted in drawings of identical sections from each rat. The repeated ANOVA showed that the between-group effects (aCSF vs. LPS) were significant (F (1, 6) = 11.24, *p* < 0.05). The regional differences (CA1, CA3, and DG) effects and interaction effects of group and region were significant (F (2, 12) ≥ 12.68, *p* < 0.05).

### Reduction of GluN2A and GluN2B subunits of NMDARs in the hippocampus of chronically LPS-infused rats

**Figure 2:**
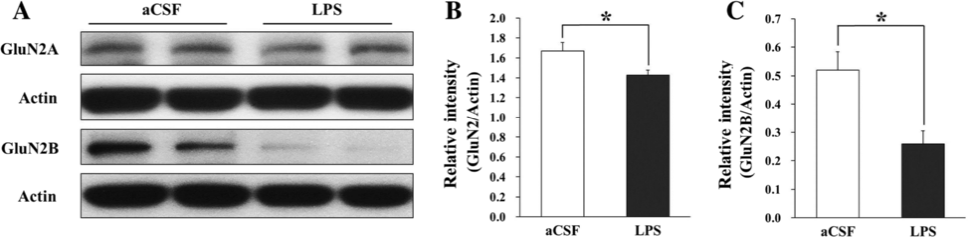
**Membrane levels of GluN2A and GluN2B subunits of NMDARs in the hippocampi of aCSF- and LPS-infused rats. (A)** Representative western blot of GluN2A, GluN2B, and actin. **(B, C)** Membrane levels of GluN2A and GluN2B were decreased in the hippocampi of the LPS-infused rats than in those of the aCSF-infused rats (*, *p* < 0.05).

The expression levels of GluN2A and GluN2B subunits of NMDARs in the membrane fraction of the hippocampus were measured using western blot. The membrane expression levels of GluN2A were slightly decreased in the hippocampi of the LPS-infused rats than in those of the aCSF-infused rats (F (1, 10) = 6.50, *p* < 0.05) (Figure 2). In addition, the membrane expression levels of GluN2B were much decreased in the hippocampi of the LPS-infused rats than in those of the aCSF-infused rats (F (1, 10) = 11.63, *p* < 0.05).

### Increased p-Erk1/2 and p-p38 in the hippocampus of chronically LPS-infused rats

**Figure 3:**
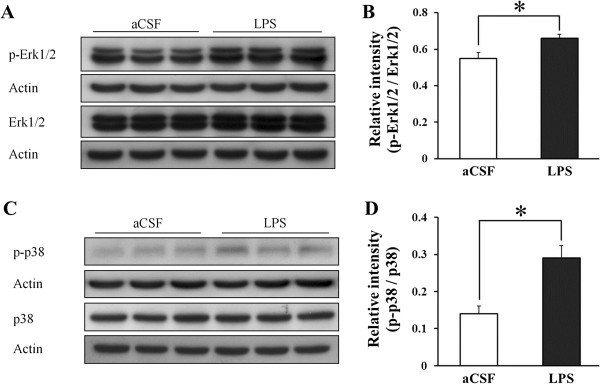
**Levels of hippocampal phosphorylated extracellular signal-regulated kinase 1/2 and phosphorylated p38 in the aCSF- and LPS-infused rats. (A)** Representative western blot of extracellular signal-regulated kinase 1/2 (Erk1/2), phosphorylated Erk1/2 (p-Erk1/2), and actin. **(B)** Levels of p-Erk1/2 were higher in the hippocampi of the LPS-infused rats than in that of the aCSF-infused rats. **(C)** Representative western blot of p38, phosphorylated p38 (p-p38), and actin. **(D)** Levels of p-p38 were higher in the hippocampi of LPS-infused rats than in that of the aCSF-infused rats. *, *p* < 0.05. The relative expression levels of all proteins were determined by densitometry and normalized to actin.

The levels of hippocampal p-Erk1/2 over p-Erk1/2 and p-p38 over p38 measured using a western blot were highly increased in the hippocampi of the LPS-infused rats than in those of the aCSF-infused rats (p-Erk1/2 (F (1, 4) = 8.55, *p* < 0.05; p-p38 (F (1, 4) = 13.48, *p* < 0.05) (Figure 3). The levels of hippocampal Erk1/2 and p38 did not differ significantly between the LPS- and aCSF-infused rats (aCSF: Erk1/2/actin [1.37 ± 0.032], p38/actin [1.47 ± 0.094]; LPS: Erk1/2/actin [1.37 ± 0.037], p38/actin [1.59 ± 0.0270]).

### Cellular distribution of p-Erk1/2 and p-p38 immunoreactivity in the hippocampus of chronically LPS-infused rats

**Figure 4:**
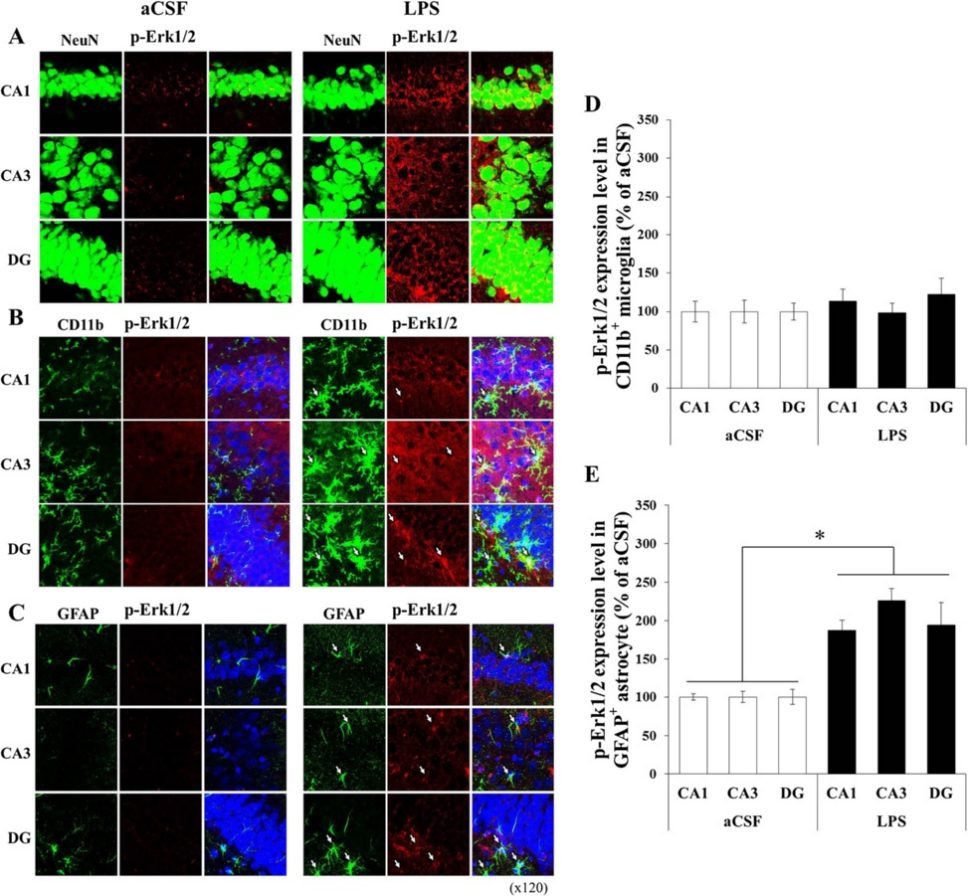
**Confocal photomicrographs of dual staining for p-Erk1/2 and markers of cellular location in the CA1, CA3, and DG of the aCSF- and LPS-infused rats.** Photomicrographs showed the overlap between p-Erk1/2-positive signals and NeuN, a neuronal marker **(A)**, or CD11b, a microglial marker **(B)**, or GFAP, an astrocyte marker **(C)**. The p-Erk1/2-positive cells were observed mostly in neurons and also in microglia or astrocytes. No differences between the LPS- and the aCSF-infused rats were observed in p-Erk1/2 levels expressing in the CD11b-positive microglia of the hippocampus **(D)**. Levels of p-Erk1/2 expressing in the GFAP-positive astrocyte were significantly increased in the hippocampal CA1, CA3, and DG areas of the LPS-infused rats compared with the aCSF-infused rats **(E)**. *, *p* < 0.01.

**Figure 5:**
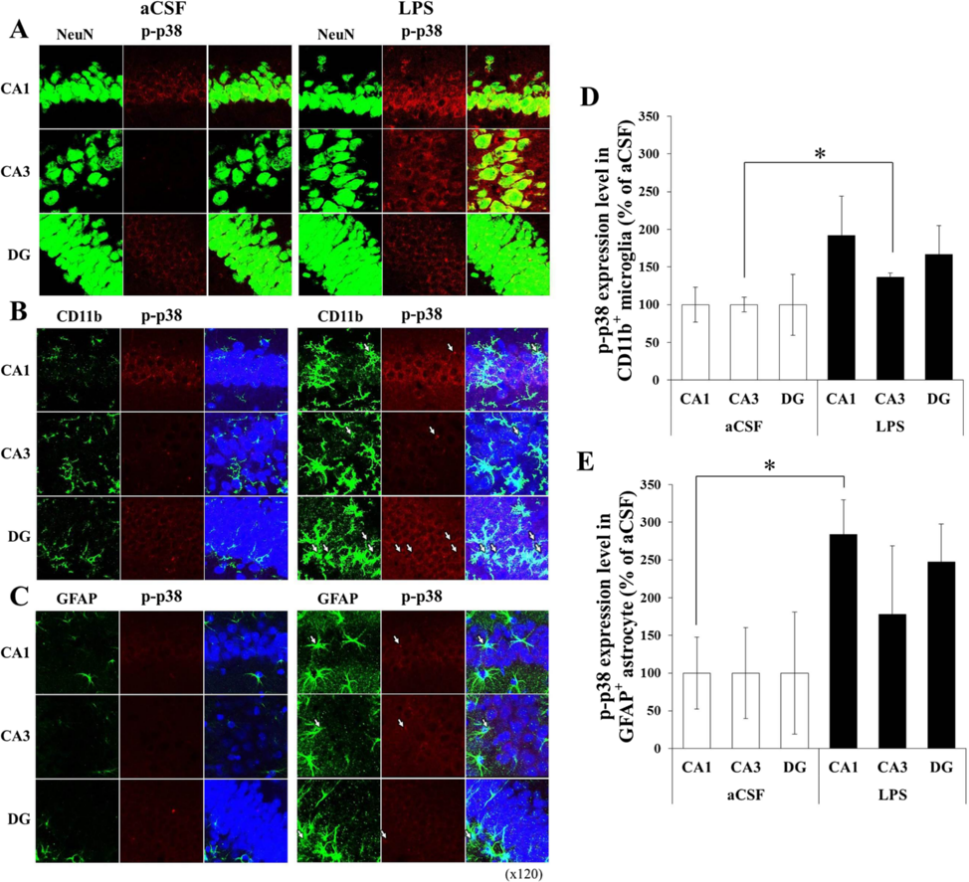
**Confocal photomicrographs of dual staining for p-p38 and markers of cellular location in the CA1, CA3, and DG of the aCSF- and LPS-infused rats.** Photomicrographs showed an overlap between p-p38-positive signals and NeuN, a neuronal marker **(A)**, or CD11b, a microglial marker **(B)**, or GFAP, an astrocyte marker **(C)**. The p-p38-positive cells were observed mostly in neurons and also in glial cells. Compared with the aCSF-infused rats, levels of p-p38 expressing in the CD11b-positive microglia were increased only in the hippocampal CA3 region of the LPS-infused (**D**; *, *p* < 0.05) and levels of p-p38 expressing in the GFAP-positive astrocyte were significantly increased only in the hippocampal CA1 region of the LPS-infused rats (**E**; *, *p* < 0.01).

To determine the cellular types that express p-Erk1/2 and p-p38 in the hippocampus of the LPS-infused rats, immunohistochemical staining was performed in the aCSF-infused rats and the LPS-infused rats. While p-Erk1/2 and p-p38 immunoreactivities were weak in the hippocampus of the aCSF-infused rats, they were strong in that of the LPS-infused rats. The p-Erk1/2- and p-p38-positive signals were located in the pyramidal cells in the hippocampal CA1, CA3, and DG areas (Figures 4 and 5). Double labeling with p-Erk1/2 and NeuN (Figures 4 and 5) demonstrated that almost all of p-Erk1/2 positive cells in the hippocampal CA1, CA3, and DG areas were neurons. The immunoreactive products were located in the cytoplasm and processes of the neurons. On the other hand, double staining with p-Erk1/2 and CD11b directed against complement receptor 3 on macrophage and microglia or anti-glial fibrillary acidic protein (GFAP) expressed in astrocytes demonstrated that p-Erk1/2 and p-p38 were also increased in glial cells. Specifically, no significant differences between the LPS-infused rats and the aCSF-infused rats were observed in p-Erk1/2 levels which were expressed in the CD11b-positive microglia of the hippocampus (Figure 4D). However, p-Erk1/2 levels of which were expressed in the GFAP-positive astrocyte were significantly higher in the hippocampal CA1, CA3, and DG areas of the LPS-infused rats than those of the aCSF-infused rats (F (1, 8) ≥ 9.34, *p* < 0.01; Figure 4E). And expression levels of p-p38 in the CD11b-positive microglia of the LPS-infused rats were significantly increased only in the hippocampal CA3 region, compared with those of the aCSF-infused rats (F (1, 4) = 10.38, *p* < 0.05; Figure 5D). Levels of p-p38 which were expressed in the GFAP-positive astrocyte of the LPS-infused rats were significantly increased only in the hippocampal CA1 region compared with those of the aCSF-infused rats (F (1, 8) = 11.00, *p* < 0.01; Figure 5E).

In the present study, rats that received chronic LPS infusion into the 4^th^ ventricle for 28 days showed poor performance on the spatial memory version of the Morris water maze task. The hippocampus responsible for spatial memory showed microglial activation, as evidenced by the increased number of Iba-1- and OX-6- positive cells. These results have been previously reported in studies using the same animal model [8,20]. In addition, electrophysiological experiments measuring synaptic plasticity such LTP and LTD in the hippocampus of chronically LPS-infused rats revealed that chronic LPS infusions impaired the induction of both NMDAR-dependent and NMDAR-independent LTP as well as NMDAR-dependent LTD in hippocampal Schaffer collateral-CA1 synapses [13,14].

In addition to impaired synaptic plasticity induced by chronic LPS infusion [13,14,24], the loss of GluN1 NMDARs was observed in the hippocampus of chronically LPS-infused rats [10]. Therefore, we predicted that chronic LPS infusion would decrease levels of GluN2A and GluN2B subunits of NMDARs and induce alteration of MAPK signaling, such as Erk1/2 and p38, associated with these subunits [16,17,25,26]. As predicted, chronic LPS infusion decreased levels of GluN2A and GluN2B in the hippocampal membrane fraction and increased phosphorylation of Erk1/2 and p38 associated with GluN2A and GluN2B.

Specifically, GluN2A and GluN2B levels of membrane fraction were much decreased, and phosphorylated Erk1/2 levels were increased in the hippocampus of rats chronically infused with LPS. The increased phosphorylation of Erk1/2 associated with decreased levels of GluN2B can be explained by the findings of a previous study, which demonstrated, using the primary cultured hippocampal cells, that GluN2B is associated with inhibition rather than activation of Ras-Erk1/2 [16]. However, there is a possibility that in response to chronic LPS infusion, the phosphorylation of Erk1/2 was increased due to reductions of other NMDA-subtype receptors such as GluN1 and GluN2A or changes in the other receptor or molecules that have not been examined so far. Chronic LPS infusion also increased levels of p-p38 in the hippocampus. It has been reported that the effects of NMDARs on p38 phosphorylation depend on the subtype composition or localization [17]. In addition, the present study examined the cellular localizations of p-p38 and p-Erk1/2 in the hippocampus of rats chronically infused with the LPS. Immunoreactivities of p-p38 and p-Erk1/2 were mostly observed in neurons of the hippocampi of chronically LPS-infused rats. However, levels of p-Erk1/2 signals expressing in the GFAP-positive astrocytes were significantly higher in chronically LPS-infused rats than aCSF-infused rats. Levels of p-p38 signals expressing in the CD11b-positive microglia and levels of p-p38 signals expressing in the GFAP-positive astrocytes were increased in the hippocampal CA1 and CA3 area, respectively, in chronically LPS-infused rats compared with aCSF-infused rats. Thus, because increased MAPK signal was also observed in the glial cells of the hippocampus with the chronic LPS infusion, it needs further investigation to draw a conclusion that chronic inflammation causes NMDAR reduction leading altered MAPK signing in neuron.

Chronic inflammation is a key feature in the pathogenesis of neurodegenerative disorders including AD [1]. Classic neuropathological features of AD include amyloid plaques and neurofibrillary tangles [3]. Clumps of activated microglia and reactive astrocytes appear in the vicinity of senile plaques [3]. Microglial cells are activated in response to exposure to LPS, interferon-*γ*, or *β*-amyloid [27,28]. In adult brain, LPS binds to microglia through toll-like receptor 4, the LPS receptor [29]. Once chronically activated, the microglial cells produce a variety of proinflammatory mediators and potentially neurotoxic compounds such as interleukin (IL)-1*β*, IL-6, tumor necrosis factor-*α*, reactive oxygen species, and nitric oxide and control neuronal activity dynamically and closely associated neurons and astrocytes [29]. Under pathological conditions, these have deleterious effects on neuronal function [30].

Through a series of animal experiment with chronic LPS infusion into the 4^th^ ventricle, the sequence of events from activation of astrocyte and microglia to alterations of MAPK signaling in the hippocampus has been examined. Chronic LPS infusion induces the release of inflammatory cytokines by activated astrocytes and microglia [31]. Subsequently, these cytokines increase the production of other inflammatory mediators such as prostaglandins [32]. Prostaglandins, in turn, induce the release of glutamate from astrocytes [33,34], resulting in an increase of extracellular glutamate and the overstimulation of glutamate receptors that lead to the loss of NMDARs and the alteration of intracellular signaling such as MAPK in hippocampal neurons [10].

A growing body of evidence suggests that inflammation is widely linked with neurodegenerative disorders including AD. As an experimental animal model of AD and other neurological disorders associated with neuroinflammation, we slowly infused LPS into the 4^th^ ventricle of Fischer-344 rats for 28 days to induce chronic inflammation. The rats with chronic LPS infusion had activated microglia, which play a role as immune effectors in the central nervous system. We also observed high levels of p-p38 and p-Erk1/2 in the hippocampus of rats chronically infused with LPS. Even though in the present study, MAPK alterations occurred mostly in neurons, we could not rule out the possibility that MAPK alterations would mainly occur in astrocytes and microglia during chronic inflammatory processes, because our follow-up period was only 4 weeks after the initiation of LPS infusion. The Aβ peptide, one of the pathologic hallmarks in AD, has been identified in the MAPK cascade as the major transduction pathway involved in the transcription of inflammatory mediators. Some studies have reported that β-amyloid fibrils in microglia stimulate rapid, transient activation of p38, resulting in inflammatory gene expression and upregulation of pro-inflammatory cytokines [35,36]. Thus, in chronic LPS animal model, chronically activated microglial cells in which phosphorylation of MAPKs occurred would produce a variety of proinflammatory mediators, leading to either the release of extracellular glutamate from glia or the functional loss of glutamate reuptake in glia. The increase of extracellular glutamate might downregulate the glutamate receptors associated with MAPK signaling.

## Conclusions

Rats chronically infused with LPS showed hippocampus-dependent spatial memory impairments in the Morris water maze task and microglial activation in the hippocampus. Levels of hippocampal p38 and Erk1/2 phosphorylation were higher in LPS-infused rats than in aCSF-infused rats, and these increases in levels were observed in neurons and in glial cells. Alteration of neuronal or glial MAPK intracellular signaling in response to inflammatory stimuli may be related to the cognitive deficits observed in AD.

## Methods

### Animals

Thirty-seven male Fischer-344 rats (SLC Inc., Shizuoka, Japan) were housed singly in colony rooms with a 12-h light/dark cycle (lights on at 0700). The rats had free access to food and water in a temperature-controlled room (22°C). The Institutional Animal Care and Use Committee of Konkuk University approved all protocols described in the report. The behavioral testing was performed during the light phase.

### Surgery

The rats were anesthetized with isoflurane and placed in a stereotaxic frame (Kopf Instruments, Tujunga, CA, USA) fitted with an isoflurane gas anaesthesia system, and an incisor bar was set at 3.3 mm below the ear bars. The scalp was incised and retracted, and holes were drilled at the appropriate locations in the skull with a dental drill. The coordinates for the 4^th^ ventricle infusions were as follows: 2.5 mm posterior to lambda on the midline and 7.0 mm ventral to the dura. Either LPS (Sigma, prepared from *Escherichia coli*, serotype 055:B5) or aCSF was chronically infused (0.25 μl/h for 28 days) through a cannula that was implanted in the 4^th^ ventricle of the brain and attached to an osmotic minipump placed under the axillary skin (model 2004; Alzet, Palo Alto, CA, USA,) as described elsewhere [10]. The composition of the aCSF (in mM) was 148 NaCl; 3.0 KCl; 1.4 CaCl_2_ 2.H_2_O; 0.8 MgCl_2_.6H_2_O; 0.8 Na_2_HPO_4_.7H_2_O; 0.2 NaH_2_PO_4_.H_2_O; pH 7.4.

### Apparatus

The rats were trained in a Morris water maze. The maze was a round tank, 1.83 m in diameter and 58 cm deep, filled to a depth of 35.5 cm with tepid (27°C) water that was made opaque by adding white paint (tempera). A moveable circular platform, 12 cm in diameter, was located 2 cm below the surface of the water. The maze was surrounded with white curtains on which 3 black cloth pieces of different shapes and sizes were attached for providing visual stimuli. A camera located above the center of the maze relayed images to a videocassette recorder and an HVS Image Analysis Computer System (Hampton, United Kingdom). Data from the water maze trials were analyzed with software provided by HVS.

### Behavioral procedure

In a standardized procedure that required the use of distal cues in a maze environment, the rats (aCSF, n = 11; LPS, n = 12) were trained to learn the position of a camouflaged escape platform [37]. Briefly, the rats were trained in 2 or 3 trials per day with an intertrial interval of 60 s, and this was performed for 9 consecutive days. The location of the platform remained constant and, in each training trial, the rats swam for 90 s or until they found the platform. Across the trials, the starting location varied among 4 equidistant points around the perimeter of the apparatus. A probe trial (every sixth trial) was conducted 24 h after every fifth training trial to assess the development of spatial bias in the maze: thus, the entire training procedure included 4 probe trials for each rat. During these probe trials, the rats swam with the platform retracted to the bottom of the pool for 30 s. After that, the platform was raised to its normal position for completion of the trial. At the completion of the protocol with the hidden platform, all rats were assessed for cue learning using a visible platform. The location of this platform varied from trial to trial in a single session of 6 training trials.

### Western blot analysis

Three rats from each group were euthanized by rapid decapitation after the behavioral experiments. The brains were removed and the hippocampi were rapidly dissected and frozen at 80°C for protein studies. Membrane isolation and protein analyses of the GluN2A and GluN2B subunits of NMDARs were extracted in the following manner: The hippocampi of the rats were dissected and homogenized in cold buffer A containing 250 mM sucrose, 20 mM HEPES (pH 7.5), 50 mM KCl, 2 mM ethyleneglycoltetraacetic acid (EGTA), and protease inhibitor cocktail (Calbiochem). The lysates were centrifuged at 800 × *g* for 10 min to remove the nuclei and large cell debris, and the supernatants were pooled and centrifuged at 100,000 × *g* for 1 h. The membrane pellet were resuspended in buffer B containing 20 mM HEPES (pH 7.0), 150 mM KCl, 2 mM EGTA, 1% (w/v) CHAPSO (Sigma), and protease inhibitor cocktail and then incubated at 4°C for 1 h with vortexing in every 5 min. The solubilized membranes were centrifuged at 100,000 × *g* for 1 h, and the supernatants were collected [38]. Proteins for the analysis of MAPKs and phosphorylated form of MAPK were extracted in the following manner: Individual tissue samples were weighed and then homogenized in 500 μl of ice-cold buffer containing 20 mM tris (hydroxymethyl) aminomethane–HCl (pH 7.4), 1% Triton-X 100, 1.5 mM EGTA, 40 mM KCl, 5% glycerol, 0.5 mM dithiothreitol, 1 mM NaF, 1 mM Na_3_VO_4_, 1 mM phenylmethylsulfonyl fluoride, and proteinase inhibitor. The homogenates were centrifuged at 18,300 × g for 60 min at 4°C.

The supernatants of the membrane fraction and the homogenates were obtained from each sample, and aliquots were taken to determine the total protein concentration using the Bradford Reagent. The proteins were then separated by sodium dodecyl sulfate-polyacrylamide gel electrophoresis and transferred to a polyvinylidene fluoride membrane. The membrane was blocked with non-fat skim milk and incubated with primary antibodies (Abs) against Erk1/2, p-Erk1/2, p38, p-p38 (1:1000, Cell Signaling), GluN2A (NR2A) (1:1000, Thermo Scientific), and GluN2B (NR2B) (1:1000, Millipore), and followed by incubation with HRP-conjugated secondary Ab (Cell Signaling). The membranes were visualized using an ECL system and developed on Hyperfilm (Amersham). The relative expression levels of all proteins were determined by densitometry and normalized to actin.

### Immunohistochemistry

After the behavioral experiments, 8–9 rats from each group were euthanized by an overdose of ketamine HCl (30 mg/kg) and xylazine (2.5 mg/kg). These rats were then intracardially perfused with 4% paraformaldehyde (PFA) in 0.01 M phosphate buffer (pH 7.4). After fixation, the brains were removed and postfixed in PFA (48 h), cryoprotected in phosphate-buffered saline (PBS) containing 30% sucrose (72 h), frozen on powdered dry ice, and sectioned (coronal plane: 40 μm) with a microtome. All sections were kept in a cryoprotectant (30% ethylene glycol, 25% glycerol, 25 mM phosphate buffer) and stored at −20°C for further use. The monoclonal antibody anti-OX-6 (1:1000, BD Bioscience) and anti-Iba-1 Ab (1:1000, Wako) was used to visualize activated microglial cells and whole microglial cells/macrophage [21,39]. The expression of Iba-1, a 17-kDa EF-hand protein, is restricted to microglia/macrophages [21]. For OX-6 and Iba-1, endogenous peroxidases were blocked by 30 min incubation in 3% H_2_O_2_/10% MeOH in PBS. The sections were incubated for 1 h at room temperature in PBS with 0.3% Triton-X (PBST) containing 10% fetal horse serum (GIBCO) and then incubated for further 18 h at 4°C in the same solution containing the appropriate primary antibody. Thereafter, sections were incubated for 1 h in the appropriate biotinylated secondary antibody at room temperature (1:200), and for 1 h in ExtrAvidin peroxidase conjugate (1:1000, Vector Laboratories). They were then reacted by means of a Vector SG substrate kit for peroxidase (Vector Laboratories). Sections were mounted onto resin-coated slides, dehydrated through ascending concentrations of ethanol, defatted in xylene, and coverslipped with Permount (Fisher).

The Iba-1-positive cells were counted to quantify all microglial cells. Sections containing the hippocampus from 4 rats per group were analyzed. For a quantitative analysis, we selected specific regions in which neuroinflammatory changes have been reported in the literature [40]. The hippocampus, including CA1, CA3, and DG, was the focus for quantification of microglial activation. One region of interest (ROI) of 0.03 mm^2^ per section in the hippocampal subregions, CA1, CA3, and DG (bregma −3.00 mm to −4.00 mm; 6 sections per rat) was selected. The number of Iba-1 microglial cells in each ROI was counted and averaged. The activated microglia had characteristic bushy morphologies with increased cell body size and contracted and ramified processes [41]. To quantify the activated microglia induced by chronic LPS infusion, the number of OX-6 positive microglia within the hippocampi from each rat were counted in drawings of identical sections.

### Double immunofluorescence

The sections were gently washed in PBST for 3 times and blocked with 5% fetal horse serum diluted in PBST for 1.5 h at room temperature, followed by treatment with a cocktail of 2 antibodies from anti-p-Erk1/2 (1:300, Cell Signaling), anti-p-p38 (1:300, Cell Signaling), anti-NeuN (1:2000, Millipore), anti-CD11b (1:1000, Serotec), and anti-glial fibrillary acidic protein (GFAP; 1:1000, BD Bioscience) antibodies for 20 h at room temperature. The sections were then treated with Alexa® 488-labeled donkey anti-mouse IgG and Alexa® 568-labeled donkey anti-rabbit IgG (1:200, Invitrogen) cocktails for 3 h at room temperature in the dark. Exposure to light was reduced to minimum after mounting and coverslip, antifade reagent (ProLong® Gold, Invitrogen) was used to maintain fluorescence until microscopy. To assess the pERK and pp38 expression level in the microglia and astrocyte, the intensity of pERK and pp38 were measured on a fluorescent microscopic picture of 400× magnifications. Region of interest (ROI) in the hippocampus were drawn along the CD11b-positive microglia and GFAP-positive astrocyte. Intensity of each ROI was measured by Fluoview 10 (Olympus, Japan).

### Statistical analysis

ANOVA and one-way repeated ANOVA were conducted to assess the impairment of spatial memory and the microglial activation (Iba-1- and OX-6-positive cell counts) induced by chronic LPS infusion, and the levels of MAPK expression (Erk1/2, p-Erk1/2, p38, and p-p38), and the levels of expression of GluN2A and GluN2B subunits of NMDARs induced by chronic LPS infusion. *P* values less than 0.05 were considered significant, unless otherwise specified.

## Abbreviations

Abs: Antibodies; aCSF: Artificial cerebrospinal fluid; AD: Alzheimer’s disease; CA1: Cornu ammonis 1; CA3: Cornu ammonis 3; DG: Dentate gyrus; Erk1/2: Extracellular signal-regulated kinases ½; GFAP: Glial fibrillary acidic protein; EGTA: Ethyleneglycoltetraacetic acid; Iba-1: Ionized calciumbinding adaptormolecule 1; LPS: Lipopolysaccharide; LTD: Long-term depression; LTP: Long-term potentiation; MAPKs: Mitogen-activated protein kinases; NMDA: *N*-methyl-D-aspartate; NMDARs: NMDA receptors; PBS: Phosphate-buffered saline; PBST: PBS with 0.3% Triton-X; p-Erk1/2: Phosphorylated Erk1/2; p-p38: Phosphorylated p38.

## Competing interests

The authors declare that they have no competing interests.

## Authors’ contributions

JM participated in animal treatments, brain dissection, water maze task, western blot analysis, immunohistology of microglial cells, data analysis, and preparation of the manuscript. BRC performed double immunostaining and immunohistology of microglial cells. CC, SSM, and WKJ participated in data processing and analysis, and organizing and drafting the manuscript. JSH conceived the study and participated in the design of the study, data processing and analysis, and organizing and drafting the manuscript. All authors have read and approved the final submitted version of this manuscript.

## Supplementary Material

Additional file 1: Figure S1 Microglial activation in the hippocampi of aCSF- and LPS-infused rats. (A) Representative photomicrograph of Iba-1-positive cells in the cornu ammonis 1 (CA1), CA3, and dentate gyrus (DG) of the hippocampus. (B) The number of hippocampal Iba-1-positive microglial cells/macrophages was higher in all subfield areas in the LPS-infused rats (LPS) than in those of the aCSF-infused rats (aCSF). (C) Representative photomicrograph of OX-6-positive cells in the hippocampus. (D) The rats with chronic LPS infusion into the 4^th^ ventricle showed highly activated OX-6-positive microglial cells in the hippocampal CA3 and DG area compared with that of the rats infused with aCSF. *, *p* < 0.05. Click here for file
