# Supplementary figures and images for: Chronic brain inflammation causes a reduction in GluN2A and GluN2B subunits of NMDA receptors and an increase in the phosphorylation of mitogen-activated protein kinases in the hippocampus
Source: Mol Brain. 2014 Apr 24;7:33. doi: 10.1186/1756-6606-7-33 (PMC4021635; doi:10.1186/1756-6606-7-33)

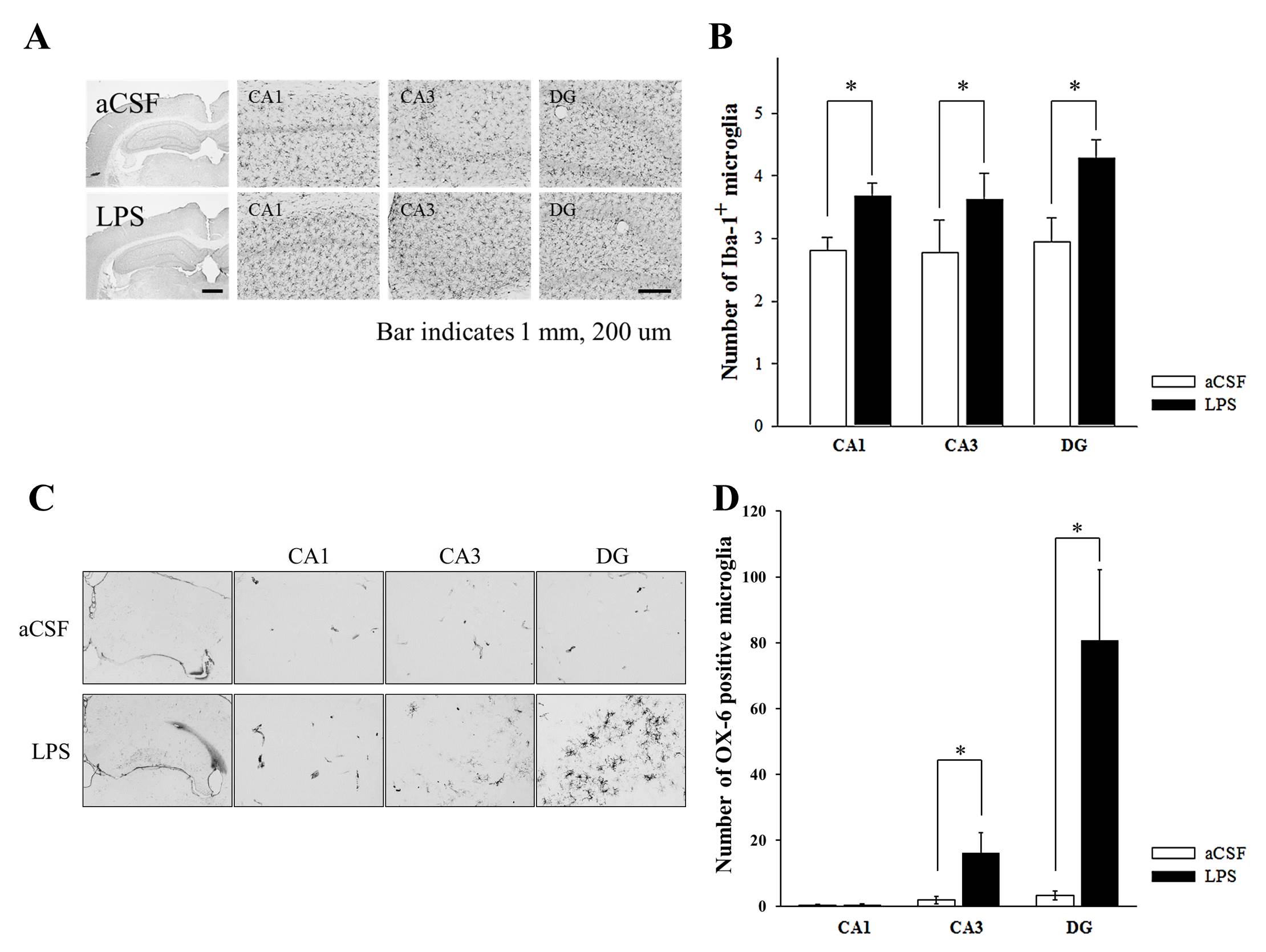

Supplement: Additional file 1: Figure S1 — Microglial activation in the hippocampi of aCSF- and LPS-infused rats. (A) Representative photomicrograph of Iba-1-positive cells in the cornu ammonis 1 (CA1), CA3, and dentate gyrus (DG) of the hippocampus. (B) The number of hippocampal Iba-1-positive microglial cells/macrophages was higher in all subfield areas in the LPS-infused rats (LPS) than in those of the aCSF-infused rats (aCSF). (C) Representative photomicrograph of OX-6-positive cells in the hippocampus. (D) The rats with chronic LPS infusion into the 4th ventricle showed highly activated OX-6-positive microglial cells in the hippocampal CA3 and DG area compared with that of the rats infused with aCSF. *, p < 0.05. [file 1756-6606-7-33-S1.jpeg]
